# Quality Evaluation of Iranian Honey Collected from Khorasan Province, Iran

**DOI:** 10.1155/2022/3827742

**Published:** 2022-02-12

**Authors:** Asma Afshari, Mahdi Ram, Sara Mohamadi

**Affiliations:** ^1^Department of Nutrition, Faculty of Medicine, Mashhad University of Medical Sciences, Mashhad, Iran; ^2^Department of Food Hygiene and Aquaculture, Faculty of Veterinary Medicine, Ferdowsi University of Mashhad, Mashhad, Iran; ^3^Department of Food Hygiene and Quality Control, Faculty of Veterinary Medicine, Shahrekord University, Shahrekord, Iran

## Abstract

Honey is a prominent nutritional and medicinal production of honey bees, originating from the nectar of flowers. The physicochemical properties of honey serve as indicators of its freshness and originality. The current survey aimed to assess the parameters of quality control, including hydroxymethylfurfuraldehyde [HMF], reducing sugars, fructose/glucose, sucrose, proline content, distaste activity, and free acidity, in 25 honey samples of different brands available in Khorasan Province, Iran. We used the methods suggested by the Association of Official Analytical Collaboration (AOAC, 1995), the International Honey Commission (IHC, 2009), and the Codex Alimentarius Honey Standards for the study. Statistical analysis was performed in Microsoft Excel. The obtained data indicated that eight out of 25 analyzed samples (32%) complied with all the requirements and were generally of acceptable quality. Meanwhile, 17 samples (68%) were unconfirmed by the Iranian Standard Organization (ISO), including 12 samples with a low level of diastase (<8 Schade) and high levels of HMF (>15 mg/kg), two samples with high sucrose levels, two samples with high proline, and one sample with high HMF. These findings suggested their inappropriate storage (time/temperature), heat treatment, and/or adulteration with industrial sugar. According to the results, the examined honey samples produced in Khorasan Province were not of acceptable quality, which highlights the importance of an effective regulatory framework to be evaluated and rectified periodically and accurately to maintain consumer rights, as well as public health.

## 1. Introduction

Honey is an aqueous natural sweetener derived from the nectar of flowers by honey bees (*Apis mellifera*) and is consumed both as a food source and a medicine [1, 2]. Honey mainly consists of 80% sugars (35% glucose, 40% fructose, and 5% sucrose) and 20% water [3, 4]. Other minor ingredients are also found in honey, such as organic acids, enzymes, amino acids, minerals, vitamins, pollen grains, and phenolic compounds [4, 5]. The most important enzymes in honey are glucose oxidase, diastase, and invertase [6]. In addition, honey contains a few quantities of amino acids, the most important of which is proline [7].

Iran is one of the most important honey-producing countries worldwide. According to the data released by the Food and Agriculture Organization of the United Nations (FAOSTAT), Iran was among the 10 largest honey-producing countries during 1993-2018 (FAOSTAT, 2018) [8]. However, several researchers have investigated the quality of Iranian honey, concluding a lack of compliance with the international standards [8, 9]. The reason is the lack of a periodical and accurate monitoring program authenticated by authorized legislations.

The quality of honey is entirely associated with its physicochemical properties, such as the content of moisture, ash, reducing/nonreducing sugars, free acidity (FA), hydroxymethylfurfural (HMF), starch, sucrose, proline, and distaste activity [10]. These properties are often measured by the International Honey Commission (IHC) [11] and the Iranian Standard Organization (ISO) [12]. Physiochemical properties could change depending on the plant source, bee variety, environmental conditions, and storage duration, as well as the harvesting and postharvest conditions [6, 13].

Fresh honey is commonly heated to suspend crystallization, eliminate microorganisms, and decrease viscosity. However, excessive heat treatment may result in the production of 5-HMF and reduction of enzymatic activity.

HMF is a heterocyclic aldehyde induced by caramelization and the Maillard reaction or hexose dehydration in acidic media. The HMF value increases in honey when it is deposited in unsuitable conditions or adulterated with invert syrup. Moreover, the presence of organic acids and low water activity favor HMF formation [3, 6]. Laboratory studies have demonstrated that 5-HMF could be carcinogenic, cytotoxic, genotoxic, and mutagenic. Therefore, its toxicity is of significance and should be monitored in honey [14].

Although honey is a natural and health-promoting product, it may sustain several harmful compounds, such as HMF, which causes health complications in the consumers. Due to the high economic value of honey and its limited availability, it is an easy target for adulteration [13], especially due to poor legislation in Iran. Given the growing desire of consumers for reliable and high-ranking honey, it is essential to identify the physicochemical properties of this valuable nutrient so that the quality and originality of honey could be ensured.

To the best of our knowledge, this is the first study to investigate the physicochemical parameters of Iranian honey available in the markets of Khorasan Province. The present study was aimed at comparing physiochemical properties such as HMF, reducing sugars, fructose/glucose (F/G), sucrose, proline, distaste activity, and FA in 25 different brands of Iranian honey available on the market of Khorasan Province, Iran, based on Iranian standards.

## 2. Materials and Methods

### 2.1. Honey Samples

In this survey, 25 samples of different honey brands available on the market of Khorasan Province were obtained fresh in sterile canisters, defined by numbers, date, and place of gathering, and stored at room temperature (22 ± 3°C) in a dark place throughout the analysis. The samples were strained through a muslin cloth before analysis to remove unwanted materials such as wax sticks, comb particles, and dead bees. Following that, the samples were analyzed in triplicate using the methods suggested by the Association of Official Analytical Collaboration (AOAC), the IHC (2009), and the Codex Alimentarius Honey Standards [1, 15, 16].

### 2.2. HMF Measurement

White's spectrophotometric method was used to measure the HMF value. Initially, five grams of each honey brand was liquefied with 25 milliliters of purified water. Afterwards, 0.5 milliliter of Carrez 1 and Carrez 2 solution (1 : 1 v/v) was added to the honey solution. The final volume reached 50 milliliters with water in a volumetric flask and was filtered. The first 10 milliliters of the filtrate were removed, and five milliliters of the honey solution was added to two test tubes. Following that, five milliliters of purified water or a honey sample was added to the first tube. In addition, five milliliters of a sodium bisulfite solution (NaHSO3 0.2%) or a reference solution was added to the second tube. The tubes were transferred to 10-millimeter quartz cuvettes, and the absorbance was read at 284 and 336 nanometers using UV-visible spectrophotometer. The following equation was used to measure HMF.

HMF (mg/kg) = (Abs284–Abs336) × 149.7 × 5 × dilution factor/weight of honey sample (g).

In the equation above, Abs284 shows the absorbance at 284 nanometers, Abs336 is absorbance at 336 nanometers, and 149.7 is the constant.

### 2.3. Measurement of Reducing Sugars

Before inversion, we used the Lane-Eynon technique based on a copper reduction method for the quantification of reducing sugars. Briefly, five milliliters of Fehling's solution A and B were mixed with seven milliliters of H_2_O and 15 milliliters of honey in a 250-milliliter Erlenmeyer flask. Following that, one milliliter of 0.2% methylene blue was added to the solution as an indicator. Afterwards, titration was carried out while heating the solution until the indicator was discolored.

### 2.4. F/G Ratio Measurement

Glucose was measured through the enzymatic oxidation procedure. In addition, fructose was quantified using the following formula:

Fructose content = total reducing sugars content − glucose content.

### 2.5. Sucrose Measurement

Sucrose was measured using the following formula:

Sucrose% = [total sugar − total reducing sugars] × 0.95.

The amount of total sugar was measured using the Lane-Eynon technique before and after inversion. Briefly, 50 milliliters of honey was added to two milliliters of diluted HCl in a 100-milliliter volumetric flask while heating in a water bath at the temperature of 70°C for 10 minutes. After cooling, phenolphthalein was added to the solution as an indicator, and titration was performed by NaOH. Once the volume reached the sign, the Lane-Eynon process continued for the solution.

### 2.6. Proline Measurement

In order to measure the proline content, five grams of the honey samples was liquified separately in 50 milliliters of water and filtered. Following that, 0.5 milliliter of the sample solution was placed inside a tube, 0.5 milliliter of water (blank test) was poured into a second tube, and 0.5 milliliter of proline standard solution was poured into three other tubes. Afterwards, one milliliter of formic acid and one milliliter of ninhydrin solution were added to each tube. The tubes were capped, shaken strongly for about 15 minutes, placed in a boiling water bath for 15 minutes, and transferred to a water bath with the temperature of 70°C for 10 minutes. At the next stage, five milliliters of propanol-water solution (1 : 1) was added to each tube at specific times. After 45 minutes, the mixture was removed from the water bath, and the absorbance was read at 520 nanometers using a spectrophotometry apparatus.

### 2.7. Diastase Number

Diastase was measured using an enzymatic-spectrophotometric approach and the Phadebas Amylase test kit (Pharmacy and Upjohn Diagnostic AB). The applied substrate was an insolvable blue-tinted cross-linked type of starch (Phadebas, Magle Life Science, USA). It was hydrolyzed by the enzyme in honey and produced blue water-soluble fractions, which were defined using a spectrophotometer at 620 nanometers. In addition, the diastase activity of the honey solution was precisely in accordance with the absorbance of the sample.

### 2.8. FA Measurement

At this stage, 10 grams of each sample was liquefied in 75 milliliters of CO_2_-free water in a 250-milliliter container. Following that, the phenolphthalein solution was added as an indicator and stirred using a magnetic stirrer. Titration was carried out using a sodium hydroxide solution (0.1 N) to the pH 8.3. The pH was determined using a pH meter (Mettler Toledo). To calculate the FA value, 10 grams of the honey solution was titrated with 10 times the volume of 0.1 M NaOH and expressed as milliequivalents of acid per kilogram of honey (meq/kg).

### 2.9. Statistical Analysis

Data analysis was performed in Microsoft Excel Statistical Package to calculate the means, percentages, and range values. The obtained data were expressed as the mean value of the triplicate laboratory tests.

## 3. Results

According to the information in Table 1, mean HMF was 35.09 mg/kg (trace: 235.3), and four honey samples (16%) had a higher HMF value compared to the legally permitted maximum level. Moreover, mean reducing sugars was estimated at 70.84% (range: 65.01-76.61%), and all the examined honey samples fulfilled the requirement in this regard. In addition, the F/G ratio was within the range of 0.900-2.800, with the mean value estimated at 1.32; all the samples complied with the standard limits of ISO in this regard (>0.900). On the other hand, mean sucrose was estimated at 4.960 g/100 g (range: 1.100-10.75%), and seven out of 25 honey samples (28%) had higher sucrose levels than the ISO maximum limit (<5%). Furthermore, mean proline was 316.1 mg/kg (range: 115.0-640.08%), and four samples (16%) had a higher value than the minimum allowable limit. Additionally, mean diastase activity was calculated to be 5.790 Schade units (range: 1-15.95 SD), and 20 samples (80%) contained a smaller number of diastases than the standard level. Finally, mean FA was estimated at 9.7 meq/kg (range: 0.00-22.00 meq/kg), and neither of the samples exceeded 40 meq/kg (legal maximum acceptable level).

## 4. Discussion

### 4.1. HMF

Honey is often processed by heating to maintain freshness, expand shelf life, decrease viscosity, and prevent crystallization [17]. However, heating may lead to the formation of compounds that do not naturally exist in fresh honey and might be harmful to human health; HMF is such an example [18].

HMF is a six-carbon heterocyclic aldehyde compound, which is considered the most important intermediate by-product in carbohydrate-containing foods, induced as a consequence of two reactions. These reactions are the degradation of hexose by acids and the decomposition of 3-deoxyosone through the Maillard reaction and/or caramelization [17, 19]. Consequently, HMF is often an indicator of honey quality, freshness, and originality [1, 2, 20]. According to the literature, HMF has genotoxic, mutagenic, and carcinogenic effects [4]. Codex Alimentarius (2001) has established a maximum value of 40.00 mg/kg for HMF in nontropical honey, as well as 80 mg/kg for tropical honey, and 15 mg/kg for honey with low enzyme levels (8-3 Schade units) [4, 21]. In addition to prolonged heat treatment and storage, several other parameters impact the formation of HMF in honey, including climatic conditions and honey composition (i.e., pH, total acidity, FA, mineral content, F/G ratio, and water activity), thereby contributing to the floral source [1, 3, 18, 22]. HMF is produced at low temperatures in acidic environments, and high temperatures along with prolonged deposition significantly increase its concentration [17, 18]. Therefore, the optimal period for the consumption of honey is within six months after harvesting [23, 24]. The rate of HMF formation also depends on the F/G ratio as it has been disclosed that fructose is five times more reactive than glucose at pH 4.6. As such, a higher F/G ratio accelerates the formation of HMF [17, 18].

According to the results of the present study, four honey samples (16%) had a higher HMF level than the legally permitted maximum level, which might be due to overheating, inadequate storage, or adulteration [16]. In another study, a higher HMF value (162.71 ± 184.94 mg/kg) was reported by Vit et al. [25]. Moreover, previous studies have shown HMF values to be within the ranges of 0-3.37 [4], 2.5-12.3 [6], 4.45-50.83 [20], 8.26-50.8 [2], 7.66-13.18 [26], 0.19-17.86 [27], and 1.10-166.25 mg/kg [28]. In a recent study in Iran [9], 5% of natural honey samples (4 out of 80) and 70% of commercial honey samples (14 out of 20) had higher HMF levels than the standard limit. In another study [29], 3.33% of the samples were reported to be unacceptable in terms of HMF content.

### 4.2. Reducing Sugars

Honey is primarily composed of simple carbohydrates such as monosaccharides (65-80%) and oligosaccharides (25%) [2]. Glucose and fructose are the most significant constituents of honey. Fructose slightly exceeds glucose [6], which could be due to the partial oxidation of glucose to gluconic acid and hydrogen peroxide by glucose oxidase [13]. The sugar composition of honey is affected by the nectar sources consumed by bees, as well as regional, climatic, and storage conditions [20, 22]. Additionally, sugar levels in honey are proportional to the maturity of honey.

During the storage and maturation of honey, sucrose gradually decomposes by the invertase enzyme supplied by bees. As a result, lower amounts of monosaccharaides and higher amounts of sucrose lead to the poor quality of honey due to an insufficient ripening period or adulteration [20]. According to the Codex Alimentarius Commission (2001), the total amount of reducing sugars (fructose and glucose) should be above 60% [16], while the ISO (No. 92, 2013) recommends levels above 65%.

In the current research, mean reducing sugars were estimated at 70.84% (range: 65.01-76.61%), and all the examined honey samples met the requirement in this regard. Our findings are consistent with the results obtained by Kamal et al. [1], Gürbüz et al. [28], and Spiric et al. [26], which indicated the value of reducing sugar in tested honey samples to be 62.55-77.25%, 68.98-75.82%, and 62.77-71.83%, respectively. On the other hand, our findings indicated slightly higher values compared to the data reported by other researchers (54.3-63.6% and 54.59-77.40%) [13, 27]. Furthermore, a lower mean value (63.3%) has been reported in indirectly adulterated (syrups feeding) samples [7]. In another study performed in Iran (Ardabil Province), the level of reducing sugars in 6.11% the samples was considered unacceptable compared to the standard level [29].

### 4.3. F/G Ratio

The F/G ratio is used as an indicator of honey crystallization, sweetness, or flavor. According to the literature, honey with higher fructose than glucose has more fluidity since fructose is comparatively more water-soluble than glucose. In addition, honey becomes sweeter since fructose is sweeter than glucose [1, 2, 22]. Honey with the F/G ratio of less than one is more likely to crystallize than remain liquefied for longer periods. Therefore, it has been confirmed that the crystallization process of honey occurs naturally rather than through adulteration [20, 22]. Notably, the actual proportion of fructose to glucose in any specific honey largely depends on the origin of the nectar [3].

According to the results of the present study, the F/G ratio was within the range of 0.900-2.800, with the mean value estimated at 1.32. All the samples complied with the standard limits of the ISO in this regard (>0.900). Consistent with our findings, the honey samples examined by Pauliuc et al. [2] were fluid as the F/G ratio was higher than one. In another research by Kamal et al. [1], the F/G ratio was reported to be 1.14-1.34. In another survey [28], the mean F/G ratio was estimated at 1.21 ± 0.15 (range: 1.03-1.67). In addition, the F/G ratios reported by de Sousa et al. [5] and Ajlouni et al. [3] were 1.1-1.5 and 1.1-1.27, respectively.

### 4.4. Sucrose

Sucrose is a sugar detected in small quantities in honey [28]. The sucrose content in honey indicates its ripening stage, botanical origin, and adulteration [13]. Overfeeding of bees with sugar in spring, adding sucrose to honey, and premature honey harvesting are common practices of honey adulteration that could increase the sucrose value [22].

In the current research, mean sucrose was estimated at 4.960 g/100 g (range: 1.100-10.75%). However, the sucrose level in seven out of 25 honey samples (28%) was higher than the ISO maximum standard limit (<5%). This range was also relatively higher than the ranges reported by Kamal et al. [1] (1.74-5.96%), Spiric et al. (2019) [26] (2.54-5.49%), Matovic et al. [27] (0.8-4.56%), and Al-Farsi et al. [13] (0-2.77%). Inconsistent with our findings, no sucrose content was detected in Saudi honey samples [22]. On the other hand, higher sucrose contents were obtained in a study performed to monitor the quality of South African honey [30], in which sucrose values were reported to be 0-32.40%. In another study [7], the researchers concluded that mean sucrose was 9.71% in indirectly adulterated samples (syrup feeding of bees), which was higher than directly adulterated samples (adding sugar syrups to pure honey; 6.38%) and the pure samples (3.79%).

### 4.5. Proline

Proline is the main amino acid found in honey, constituting approximately 59% of the entire amino acid content [19]. The major sources of proline are the salivary glands of honeybees and plants. Proline is an indicator of honey maturation and adulteration and is also used to find floral sources [13]. The permissible standard level for proline is above 180 mg/kg (ISO No. 92, 2013).

According to the results of the present study, mean proline was 316.1 mg/kg (range: 115.0-640.08). In addition, four samples (16%) had a higher proline level than the minimum allowable limit. This is consistent with another survey [1], which indicated the proline content to be within the range of 390.33-453.67 mg/kg. Similarly, mean proline was reported to be 420 ± 174 mg/kg in a recent study (range: 117.15-933.49) [28]. In Turkish honey samples, this value was reported to be 404.2-881.7 mg/kg [6], while lower levels of proline (47.6 ± 1.87 mg/kg) were reported by Belay et al. [4].

### 4.6. Diastase

Diastase is a class of enzymes that consists of *α*- and *β*-amylase and naturally exists in honey. Its activity is expressed in Schade, Goethe, or diastase units. A unit of diastase is the amount of enzyme that is able to convert 0.01 gram of starch substrate (insoluble blue-dyed starch) within one hour at the temperature of 40°C. Diastase is a common indicator of honey freshness [31]. Due to heat susceptibility, this indicator could be influenced by storage conditions (e.g., high temperatures, and prolonged storage) and decrystallization [19]. Based on international legislation (Codex Alimentarius Commission, 2001), high-quality honey should contain eight Schade units of diastase per gram of honey [19, 31].

In the current research, mean diastase activity was estimated at 5.790 Schade units (range: 1-15.95 SD). Moreover, 20 samples (80%) contained lower number of diastases and were below the standard limit. Notably, the enzyme level alone cannot be a reliable indicator of quality control unless it is supported by other quality control parameters (mainly HMF) [4]. According to the international codex, honey samples with low enzymatic levels (<8 Schade units) should be associated with lower HMF value (<15 mg/kg) to meet the quality standard [4]. Accordingly, 11 out of 20 samples with low diastase activity in the present study had higher HMF (>15 mg/kg), which suggested inappropriate storage (time and temperature), heat treatment, and/or adulteration with industrial sugar.

Inconsistent with our findings, recent studies [1, 6, 28] have reported higher ranges of diastase, estimated at 12.63-16.33, 0.00-20.60, and 6.35-18.6 Schade units. On the other hand, diastase activity in Oman honey was reported to be 5.57 Schade units (range: 1.46-18.4) for Sidr and 2.49 Schade units (range: 0.78-5.55) for multiflora samples, which is consistent with our findings [13]. Matovic et al. also reported a significantly higher range of this enzyme [27], calculated to be 29.24-60.0 Schade units. The broad range of reported diastase values could be due to different factors, such as climatic conditions, botanic origin, processing, storage conditions, bee species, and harvesting time [4, 13, 31].

### 4.7. FA

FA is significantly associated with the induction of equilibrium between organic acids and the internal esters/lactones and inorganic ions (i.e., phosphate, sulfate, and chloride). It is the principal element responsible for the taste of honey [7]. The most important organic acids are gluconic and citric acid, as well as butyric, succinic, malic, acetic, formic, pyroglutamic, and lactic acids [6]. Increased FA may indicate sugar fermentation due to inappropriate processing, high microbial load (e.g., xerotolerant yeast), early harvesting, and immature honeycombs [7, 20]. Moreover, factors such as the water content, floral sources, bee species, harvest season, and geographical origin could affect this value [5, 30].

In the present study, mean FA was estimated at 9.7 meq/kg (range: 0.00-22.00 meq/kg), and none of the samples exceeded 40 meq/kg (legal maximum acceptable level). Consistent with our findings, mean FA was reported to be within the range of 10.25 ± 0.01 − 20.34 ± 0.18 meq/kg [3]. Similarly, previous investigations have indicated the FA content to be within the ranges of 8.50-24.83 [26] and 9.31-43.7 meq/kg [27]. Meanwhile, some studies have reported higher FA values (14.94, 31.63, and 74.72 meq/kg) [2, 28, 31]. On the other hand, a lower mean FA range has been reported by Crăciun et al. [7] in indirectly adulterated honey (feeding bees with syrups; 5.5 meq/kg). As for directly adulterated honey (mixing sugar syrups with honey), this value was estimated at 5.0 meq/kg, while it was 0.75 meq/kg in pure honey in the mentioned study.

## 5. Conclusion

Physicochemical parameters are indicators of the originality and freshness of honey. Therefore, a high-grade honey is characterized by high diastase and low HMF. In the case of a low diastase value, HMF should be lower than 15 mg/kg to comply with the standard requirements. According to our findings, eight out of 25 analyzed samples (32%) complied with all the standard requirements and had acceptable quality. Meanwhile, 17 samples (68%) did not meet the ISO standards, including 12 samples with low diastase (<8 Schade) and high HMF (>15 mg/kg), two samples with high sucrose, two samples with high proline, and one sample with high HMF. These findings implied the inappropriate storage (time/temperature), heat treatment, and/or adulteration with industrial sugar. Therefore, effective regulatory framework should be implemented and rectified periodically and precisely to maintain consumer rights, as well as public health.

## Figures and Tables

**Table 1:** The physicochemical parameters of Iranian honey samples (number of tested samples = 25).

Parameters	Min.−max. values	Average	Standard limits of ISIRI	Samples exceeding limits
HMF (mg/kg)	Trace-235.3	35.09	Max. 40	4
Reducing sugars before hydrolysis%	65.01-76.61	70.84	Min. 65	0
Fructose/glucose	0.90-2.800	1.320	Min. 0.9	0
Sucrose g/100 g	1.100-10.75	4.960	Max. 5	7
Proline (mg/kg)	115.0-640.0	316.1	Min. 180	4
Diastase (Schade units)	1.00-15.95	5.790	Min. 8	20
Free acidity meq/kg	0.00-22.00	9.700	Max. 40	0

## Data Availability

The dataset used is available from the corresponding author (saramohamadi12@yahoo.com), upon judicious request.
